# Auditory neuroanatomy: a sound foundation for sound processing

**DOI:** 10.3389/fnana.2012.00048

**Published:** 2012-12-06

**Authors:** Miguel A. Merchán, Enrique Saldaña, Douglas L. Oliver

**Affiliations:** ^1^Laboratory 6, Neuroscience Institute of Castilla y León, Universidad de SalamancaSalamanca, Spain; ^2^Department of Neuroscience, University of Connecticut Health CenterFarmington, CT, USA

Auditory neuroanatomy has a long and distinguished history. Santiago Ramón y Cajal and Hans Held were pioneers who began the process of identifying the cellular components of the central auditory system. The stature of these early neurohistologists keeps growing today as the field of auditory neuroanatomy moves from the gross identification of brain structure to the analysis of neuronal morphology and function.

Auditory neuroanatomy, the topic of this special issue and E-Book, focuses on the study of the morphology of the neurons and nuclei in central auditory system and is now at the interface between cell biology, electrophysiology, and molecular biology. Because hearing is a complex phenomenon, researchers select the animal models that are best suited to investigate particular issues. Thus, the chapters of this E-Book include studies done with mice (normal and transgenic), rats, guinea pigs, cats (normal and congenitally deaf), ferrets, pallid bats, and primates. Moreover, given the morphological complexity of the nervous system, the authors of this volume have applied an arsenal of classical and state-of-the-art techniques that, alone or in daring combinations, cover the methodological spectrum found in modern neuroscience. Here, electron microscopy, histochemistry, immunocytochemistry, confocal microscopy, anterograde, and retrograde tract-tracing, surgical lesions, genetic engineering, molecular biology, *in situ* hybridization, auditory brainstem recordings, extracellular single-unit recordings, or magnetic resonance imaging are used to provide meaningful answers to pertinent questions about the morphological substrate of normal and abnormal hearing.

The E-Book begins with a detailed longitudinal study of the insulin-like growth factor-I (IGF-I) gene on hearing in wild type *Igf1*^+/+^ and null *Igf1*^−/−^ mice (Riquelme et al., 2010). Using a skilled combination of auditory brainstem recordings, *in vivo* magnetic resonance imaging, histological and immunocytochemical stains, quantitative PCR, and biochemical assays, the authors demonstrate that *Igf1*^−/−^ mice present a profound deafness at all ages studied. In contrast, *Igf1*^+/+^ mice suffer significant age-related cochlear alterations and hearing loss parallel to a decrease in circulating levels of IGF-1.

David K. Ryugo and his coworkers (Baker et al., 2010) investigate postnatal development of the endbulbs of Held (the giant synapses between the fibers of the cochlear nerve and spherical bushy cells of the ventral cochlear nucleus) in the congenitally deaf white cat. The endbulbs are present at birth, but they have an abnormal synaptic ultrastructure despite the seemingly intact cochlear structure. Because these alterations in kittens can be reversed by electric stimulation applied though cochlear implants, Ryugo's findings underscore the importance of early intervention in congenitally deaf children.

Ito and Oliver (2010) use the gene expression of vesicular glutamate transporters to identify glutamatergic neurons that send inputs to the inferior colliculus. Identification of a glutamatergic synapse is often much easier than identifying the neuronal source of the presynaptic terminal, especially when it is in a distant location. Here, the glutamatergic neurons that use the VGLUT2 transporter are identified, and these are the likely sources of the dense VGLUT2 axosomatic terminals on the large GABAergic tectothalamic neurons in the colliculus.

The article from Enrique Saldaña's laboratory (Viñuela et al., 2011) analyzes the relationships between two enigmatic nuclei: the superior paraolivary nucleus (SPON) and the tectal longitudinal column (TLC). Experiments with the bidirectional tracer biotinylated dextran amine (BDA) reveal that the rat SPON sends hitherto unknown projections to the entire ipsilateral TLC and the rostral and caudal portions of the contralateral TLC. They also show that SPON projects to the deep layers of the superior colliculus and to the periaqueductal gray matter, and that the SPON and the ipsilateral TLC are reciprocally connected.

Miguel A. Merchán's group (Clarkson et al., 2010) employs immunocytochemistry to analyze how the unilateral ablation of the cerebral auditory cortex modifies the sound-induced expression of c-Fos in the inferior colliculus of the rat. Using stereology, morphometry, and densitometry, they determined the number of labeled neurons, and the size and the intensity of the immunostaining of their nuclei at various times after injury (1, 15, 90, and 180 days). The number of immunoreactive neurons decreases for 15 days after lesions, but then there is a progressive partial recovery that suggests a successful long-range repair or adaptation in the IC.

Motts and Schofield (2010) use fluorescent retrograde tracers to show that the medial geniculate body of the guinea pig receives direct projections from neurons in the pedunculopontine tegmental nucleus (PPT) and the laterodorsal tegmental nucleus (LDT). These two nuclei are associated with arousal and control of the sleep/wake cycle. By combining retrograde tracers and immunocytochemistry for choline acetyltransferase (ChAT), they further demonstrate that approximately 50% of these neurons are cholinergic.

The study headed by Andrew J. King (Bajo et al., 2010) uses neuroanatomical tracers to investigate the projections from the auditory neocortex to the superior colliculus in the ferret. These projections arise from layer V pyramidal neurons located preferentially in the anterior ventral field (anterior ectosylvian gyrus) and in the posterior suprasylvian field (posterior ectosylvian gyrus), are predominantly ipsilateral, and end more densely in the caudal half of the superior colliculus, particularly in the posteromedial quadrant.

Razak and Fuzessery (2010) review the organization and development of auditory thalamocortical pathways in the pallid bat. Two parallel pathways extend from the inferior colliculus to the cortex and process information about echolocation calls and passive listening. These pathways become adult-like after only 2 months of postnatal development and it is suggested that they depend on experience-dependent plasticity to segregate.

Finally, the paper from Ricardo Insausti's laboratory (Munoz-Lopez et al., 2010) reviews the evidence supporting the two prevailing hypothesis for the organization of the circuits that underlie the memory for the recognition of sounds in primates. The “direct stream” circuit is made of projections from the auditory association areas of the superior temporal gyrus to the medial temporal cortex, while the “indirect stream” circuit predicts one or more synapses in intermediate, polymodal cortical areas before the information reaches the medial temporal cortex.
